# Supplementary material for: The Role of CRABS CLAW Transcription Factor in Floral Organ Development in Plants
Source: Int J Mol Sci. 2025 Sep 25;26(19):9377. doi: 10.3390/ijms26199377 (PMC12525216; doi:10.3390/ijms26199377)
Supplement: Supplementary file 1 [file ijms-26-09377-s001.zip › Description to Supplement Materials.pdf]

Fig. S1. Multiple sequence alignment of 100 CRC protein sequences reveals strongest conservation of amino acid residues within the C2C2-type zinc finger motif, the N-terminal half of the Ser/Pro-rich domain, the nuclear localization signal (NLS), and the N-terminal half of Helix 1. Fragments of highest conservation are marked in red.

Table S1.ChIP and RNA-seq studies indicate that AG regulates 1,985 genes in *A. thaliana*, of which 225 appear to be direct AG targets [42]. In addition, Uemura et al. (2018) identified 125 direct AG targets in the *A. thaliana* genome [71]. Representative genes that are either directly regulated by AG or integrated within the AG regulatory network are listed.

Table S2. Genes and proteins associated with CRC regulation and the biological outcomes of their interactions.

File S1. Detailed information concerning putative CRC functional partners characterized by the STRING database.
