# Supplementary material for: The Role of CRABS CLAW Transcription Factor in Floral Organ Development in Plants
Source: Int J Mol Sci. 2025 Sep 25;26(19):9377. doi: 10.3390/ijms26199377 (PMC12525216; doi:10.3390/ijms26199377)
Supplement: Supplementary file 1 [file ijms-26-09377-s001.zip › Fig S1 Multiple protein sequence alignment.pdf]

NCBI Multiple Sequence Alignment Viewer, Version 1.26.0

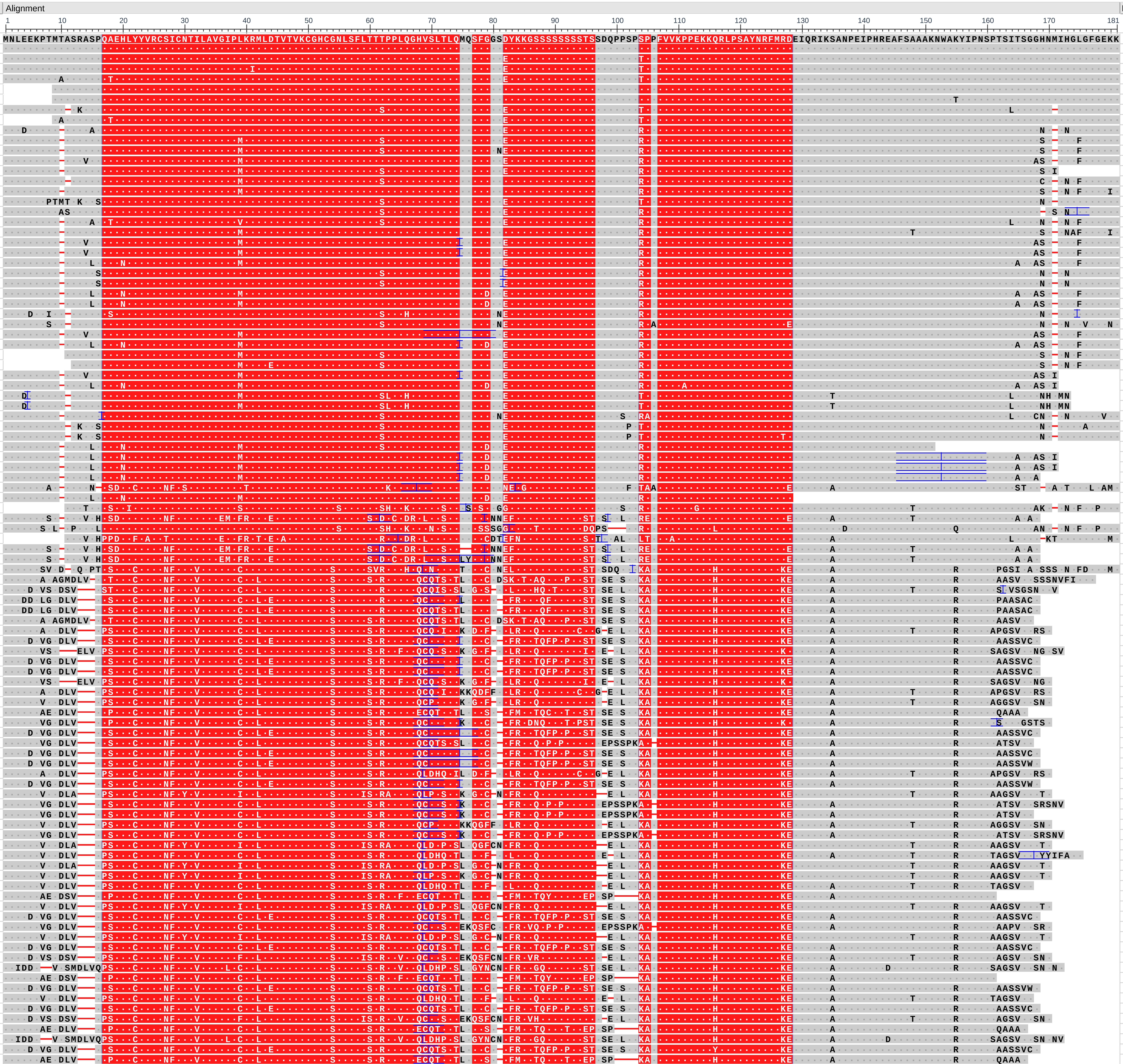

Sequence ID		Start	End	Organism
Q8L925.2	(+)	1	181	Arabidopsis thaliana
NP_177078.1	(+)	1	181	Arabidopsis thaliana
KAG7586382.1	(+)	1	181	Arabidopsis thaliana x A...
KAG7589478.1	(+)	1	181	Arabidopsis suecica
XP_002888705.1	(+)	1	181	Arabidopsis lyrata sub...
OAP14811.1	(+)	1	173	Arabidopsis thaliana
AAM66994.1	(+)	1	173	Arabidopsis thaliana
XP_006301241.1	(+)	1	179	Capsella rubella
CAE5963515.1	(+)	1	173	Arabidopsis arenosa
VVA94563.1	(+)	1	179	Arabis nemorensis
XP_018455362.2	(+)	1	179	Raphanus sativus
XP_056855915.1	(+)	1	179	Raphanus sativus
XP_009105464.1	(+)	1	179	Brassica rapa
KAL0701242.1	(+)	1	170	Brassica carinata
KAJ0241330.1	(+)	1	179	Hirschfeldia incana
KAF8082091.1	(+)	1	179	Sinapis alba
XP_010470890.1	(+)	1	180	Camelina sativa
CAA7053621.1	(+)	1	184	Microthlaspi erraticum
KFK41341.1	(+)	1	179	Arabis alpina
KAF8077069.1	(+)	1	179	Sinapis alba
VDD01664.1	(+)	1	180	Brassica rapa
CAF2192011.1	(+)	30	209	Brassica napus
KAF3520159.1	(+)	1	179	Brassica cretica
ESQ28339.1	(+)	35	214	Eutrema salsugineum
XP_024007404.1	(+)	1	180	Eutrema salsugineum
XP_013588611.1	(+)	1	179	Brassica oleracea var. ol...
XP_048617202.1	(+)	30	208	Brassica napus
CAH2066310.1	(+)	1	180	Thlaspi arvense
CAN8236022.1	(+)	1	179	Cochlearia groenlandica
KAH0919781.1	(+)	1	194	Brassica napus
KAH0875465.1	(+)	1	180	Brassica napus
KAL0801322.1	(+)	2	171	Brassica carinata
CAH8363928.1	(+)	3	171	Eruca vesicaria subsp. s...
CAN7053610.1	(+)	30	200	Brassica rapa subsp. tril...
ACG60679.1	(+)	1	170	Brassica oleracea var. a...
AAU12183.1	(+)	1	173	Lepidium africanum
AAW83049.1	(+)	1	173	Lepidium africanum
KAL1218491.1	(+)	1	180	Cardamine amara sub...
XP_010511942.1	(+)	1	179	Camelina sativa
XP_010415547.1	(+)	1	179	Camelina sativa
KAG2322664.1	(+)	1	150	Brassica carinata
CAN7087358.1	(+)	30	229	Brassica oleracea var. bo...
CAF2062942.1	(+)	30	229	Brassica napus
CAN6934384.1	(+)	30	226	Brassica oleracea
AAW83050.1	(+)	1	185	Cleomella sparsifolia
AAU12182.1	(+)	1	127	Brassica oleracea
KAL0731378.1	(+)	2	173	Brassica carinata
XP_010519367.1	(+)	1	173	Tarenaya hassleriana
KAF8096340.1	(+)	1	178	Sinapis alba
XP_010551365.1	(+)	2	178	Tarenaya hassleriana
XP_010519368.1	(+)	1	171	Tarenaya hassleriana
XP_010519366.1	(+)	1	186	Tarenaya hassleriana
AAW83045.1	(+)	1	186	Cynophalla flexuosa
OMO93524.1	(+)	1	179	Corchorus capsularis
GAV85385.1	(+)	1	171	Cephalotus follicularis
GMI75312.1	(+)	1	170	Hibiscus trionum
GMI75311.1	(+)	1	167	Hibiscus trionum
OMO72055.1	(+)	1	169	Corchorus olitorius
XP_021642286.2	(+)	1	168	Hevea brasiliensis
XP_040944534.1	(+)	1	170	Gossypium hirsutum
XP_057986043.1	(+)	1	170	Hevea brasiliensis
XP_040944535.1	(+)	1	170	Gossypium hirsutum
XP_040944533.1	(+)	1	170	Gossypium hirsutum
XP_057986047.1	(+)	1	168	Hevea brasiliensis
XP_021642285.2	(+)	1	169	Hevea brasiliensis
XP_012067815.1	(+)	1	168	Jatropha curcas
KAK8567372.1	(+)	1	165	Hibiscus sabdariffa
XVF03162.1	(+)	1	174	Reevesia pubescens
KAA3483798.1	(+)	1	175	Gossypium australe
XP_017970271.1	(+)	1	165	Theobroma cacao
KAG4158631.1	(+)	1	175	Gossypium hirsutum
TYH24950.1	(+)	1	175	Gossypium darwinii
XP_021642287.2	(+)	1	166	Hevea brasiliensis
PPR84106.1	(+)	1	170	Gossypium barbadense
XP_042981989.1	(+)	1	167	Carya illinoinensis
KAK6281008.1	(+)	1	173	Theobroma cacao
XP_017970270.1	(+)	1	168	Theobroma cacao
XP_020533631.1	(+)	1	169	Jatropha curcas
KAK6242613.1	(+)	1	173	Theobroma cacao
XP_042981990.1	(+)	1	166	Carya illinoinensis
XP_048235919.1	(+)	1	176	Ricinus communis
XP_042981992.1	(+)	1	165	Carya illinoinensis
XP_018852419.1	(+)	1	167	Juglans regia
XP_048235923.1	(+)	1	163	Ricinus communis
KAE8673250.1	(+)	1	156	Hibiscus syriacus
KAF5449494.1	(+)	1	166	Juglans regia
XP_012481221.1	(+)	1	167	Gossypium raimondii
XP_021293283.1	(+)	1	171	Herrania umbratica
XP_018852421.1	(+)	1	165	Juglans regia
XP_052483754.1	(+)	1	167	Gossypium raimondii
XP_030961618.1	(+)	1	169	Quercus lobata
XP_048318942.2	(+)	1	171	Ziziphus jujuba
XP_039034686.1	(+)	1	156	Hibiscus syriacus
KAB2090332.1	(+)	1	167	Gossypium barbadense
EEF50724.1	(+)	1	163	Ricinus communis
XP_016667797.1	(+)	1	167	Gossypium hirsutum
XP_050278441.1	(+)	1	169	Quercus robur
KAL4304300.1	(+)	1	163	Hibiscus cannabinus
KAH7516124.1	(+)	1	171	Ziziphus jujuba var. spin...
TYI36265.1	(+)	1	167	Gossypium tomentosum
KAK8699689.1	(+)	1	163	Hibiscus sabdariffa
