# Supplementary material for: The Role of CRABS CLAW Transcription Factor in Floral Organ Development in Plants
Source: Int J Mol Sci. 2025 Sep 25;26(19):9377. doi: 10.3390/ijms26199377 (PMC12525216; doi:10.3390/ijms26199377)
Supplement: Supplementary file 1 [file ijms-26-09377-s001.zip › Table S1 and S2 to v 11 of manuscr..pdf]

Table S1.ChIP and RNA-seq studies indicate that AG regulates 1,985 genes in *A. thaliana*, of which 225 appear to be direct AG targets [42]. In addition, Uemura et al. (2018) identified 125 direct AG targets in the *A. thaliana* genome [71]. Representative genes that are either directly regulated by AG or integrated within the AG regulatory network are listed.

**Genes directly regulated by AG or present within AG regulatory network**	**Biological function**	**Reference**
KNU, *CRC*, *JAGGED*, *SEPALLATA3* (*SEP3*),*NOZZLE/SPOROCYTELESS*, *SHATTERPROOF2*, *AP1*, *AP3*, *AG*, *ARABIDOPSIS THALIANA HOMEO-BOX GENE1*	C-function (stamen and carpel development)	2,42,67
PI, *SHP1*, *UNUSUAL FLORAL ORGANS*, *SUPERMAN* (*SUP*), , *HECATE1* (*HEC1*), *HEC2*, *VERDANDI*	3-rd and 4-th whorl development (stamen and ovules)	2,42,67
Chromomethylases*CMT2* and *CMT3*, the histone methyltransferase *KRYPTONITE*, the chromatin remodeler *DEFICIENT IN DNA METHYLATION1*, and the *ARGONAUTE* (*AGO*) protein family members (AGO4,6,9)	Transposon silencing through DNA cytosine methylation	2,42,67
SEPALLATA3 (*SEP3*), *APETALA3* (*AP3*), *PISTILLATA* (*PI*), and *AG*	Four genes are regulated through a coordinated positive feedback loop that sustains their own expression and promotes stamen development in whorl 3	67,68
SEPALLATA3 (*SEP3*), *AG*	Two genes *SEP3* and *AG* are regulated through a coordinated positive feedback loop that sustains their own expression and promotes carpel development, with *CRC* expression being further reinforced.	67,68
ULTRAPETALA1 (*ULT1*)	activates *AG1* independently from *LFY*. which contributes to FM termination regulation or floral meristem identity and determinacy<br>*ULT1* activates *AG1* independently of *LFY*, thereby contributing to the regulation of floral meristem (FM) termination as well as	69,70

	floral meristem identity and determinacy.	
From stage 3 onward, *SUPERMAN* regulates floral meristem size in conjunction with *AG* and *CLV3*	Regulation of floral meristem size.	71
MONOPTEROS (*MP*); *ARABIDOPSIS HISTIDINE PHOSPHOTRANSFER PROTEIN6* (*AHP6*), *TARGET OF MONOPTEROS3* (*TMO3*), *AINTEGUMENTA-LIKE6* (*AIL6*), and *LEAFY* (*LFY*).	Regulators that are negatively influenced by AG, but promote the specification of organ initial cells and the establishment of the floral meristem.	71
CRABS CLAW (*CRC*) and *SPOROCYTELESS* (*SPL*)	Examples of AG-activated targets involved in floral meristem formation.	71
S*AWTOOTH, KNATM, POUND-FOOLISH, SHOOT GRAVITROPISM5, PHYTOCHROME INTERACTING FACTOR3-LIKE1, MYB DOMAIN PROTEIN110, AUXIN RESPONSE FACTOR11, REPRODUCTIVE MERISTEM11* (*REM11*) *NAC DOMAIN CONTAINING PROTEIN79*, V*ERDANDI*, *At*1g26610, and *At*3g57370, four *MADS*-box and four *AP-2* type TFs	TFs directly regulated by AG	71
PHABULOSA, SHATTERPROOF1 (*SHP1*), *SHP2, HALF FILLED, SPL, HECATE1* (*HEC1*), *HEC2, LFY, REM11, MIR167A, ROXY1, AGAMOUS-LIKE18* (*AGL18*), *JAGGED, CRC, CUP-SHAPED COTYLEDON1, GA INSENSITIVE DWARF1B, FLOWERING LOCUS C, PROTODERMAL FACTOR 2* (*PDF2*), *LMI2, STERILE APETALA* (*SAP*), and *AIL6, PDF2, LFY, UNUSUAL FLORAL ORGANS* (*UFO*), *AIL6*, and *SAP*	AG targets involved in flower development	71
BELL1-like (BELL) homeobox protein	The age-dependent and high temperature–enhanced repression of *AG*	72

Table S2. Genes and proteins associated with *CRC*/CRC regulation and the biological outcomes of their interactions.

**Genes regulating *CRC***	**Biological outcome**	**Reference**
Among the 140 proteins interacting with the *CRC* promoter in Y1H assays, 48 were annotated in PlantPAN3. This group included several genes expressed during gynoecium development, such as *NGA2,3,4, ETT, BP, SPT, GIK, RPL, ARF4,6YAB2,3,5, NUB, PHP, ATH1, INO, Sep-03, AG, SLK1,2, ULT1, PHV, SEU, CNA, Sep-04, CRC, LUG, REV*.	Putative regulation of gynoecium development	12
GCN5 activates *AGAMOUS (AG*), *KNUCKLES (KNU*), and *CRABS CLAW (CRC*).	Regulation of floral meristem determinacy	83
In the gynoecium, CRC expression is negatively regulated by A-class genes, including *AP2* and *LUG*.	Regulation of radial growth in the gynoecium	4
In the third floral whorl, CRC expression is negatively regulated by *PI* and *AP3*.	Regulation of CRC functions in the third whorl (stamens)	4
AG	*AG* is required for optimal and spatially restricted *CRC* expression	4
GYM, KAN	Regulation of abaxial-adaxial tissues development within carpel	107
SEUSS and *AINTEGUMENTA*	*SEUSS* and *AINTEGUMENTA* act synergistically in regulating ovule formation and medial gynoecium development.	108
JAIBA (HAT1),	FM determinacy and development of the gynoecium medial domain	109
STY1	Development of stylar, stigmatic, septal, and medial xylem tissues	110
GCN5	Regulation of FM termination	83
PfDOLL1 (*PfGLO1*) negatively regulates *PfCRC* in the 2-nd and 3-rd floral whorl.	Regulation of corolla and stamen	111

	development	
PfDOLL1 (*PfGLO1*) positively regulates *PfCRC* in the 4-th floral whorl.	Regulation of carpel development	111
Gα-subunit CsGPA1 protein activates *CsCRC* transcription in cucumber.	Inhibition of floral organ numbers and promoting fruit elongation in cucumber	62
**Genes regulated by *CRC***	**Biological outcome**	**Reference**
Brassinosteroid-dependent *SlWUS* repression by *Sl*CRCa	Repression of *WUS* gene	39
The *PfCRC* negatively regulates *PfGLO2* in the 2-nd and 3-rd floral whorl.	Regulation of corolla and stamen development	111
The *PfCRC* positively regulates *PfGLO2* in the 4-th floral whorl.	Regulation of carpel development	111
Repression of *TRN2*	Formation of local auxin maxima and indirect *WUS* repression	12,77
Activation of *KCS7* and *KCS15*	VLCFA biosynthesis	100
Repression β-1,3-glucanase gene (*GLU19*) in *Gossypium barbadense* and *Gossypium hirsutum*.	Repression of *GLU19* in cotton increases seed and fibre yield in cotton	112
**Proteins interacting with CRC**	**Biological outcome**	**Reference**
CRC and INO interact (Y2H, BiFC) with a common partner, *Pg*BEL1 in *Punica granatum* (pomegranate)	Maintenance of ovule identity and seed development in *Punica granatum*	73
*Sl*CRCa and *Sl*CRCb proteins interact with each other, and both also associate with *Sl*KNU, *Sl*IMA, and *Sl*HDA1. However, only *Sl*CRCb, and not *Sl*CRCa, interacts with *Sl*TPL1	Repression of *WUS* gene	3
ARF19 (auxin responsible TF), ARR12 (cytokinin responsible TF) interact with CRC (Y2H, BiFC)	Regulation of gynoecium development.	17
TCP7,13, BIM1, NGA3 interact with CRC (Y2H, BiFC)	TFs of unknown functions in gynoecium development control.	17
Analysis of chimeric INO–CRC proteins showed that the amount of CRC included in the chimera had a quantitative effect on the protein'stability to complement *crc-1*.<br>Contrary to the *crc* complementation analysis, replacement of either the variable or carboxyl-terminal regions of *INO* with those of *CRC* resulted in relative insensitivity to inhibition by *SUP*. Only the amino-terminal exchange retained the ability to both promote integument growth and respond appropriately to *SUP*, albeit at a reduced frequency.	Regulation of CRC functions in nectary and carpel formation	113
TCP4 and TCP18 interact with CRC (Y2H) to putatively regulate *NGA* gene expression. The *NGA* genes, in turn,	Inhibition of style elongation, regulation of	114

enhance *SSS2* expression, leading to inhibition of style elongation. TCP4 and CRC also independently regulate *YUC* genes, while both can form multimeric complexes with NGAs to cooperatively regulate auxin biosynthesis and establish the identity of the apical gynoecium.<br>The interaction between CRC and TCP4 was confirmed in plant, using Luciferase complementation imaging (LCI) assays to show a clear fluorescence in the *N. benthamiana* leaves that co-expressed 35S-CRC-nLUC and 35S-cLUC-mTCP4, while no fluorescence was detected in the control combinations.<br>TCP5 and TCP12 relatively weakly interact with CRC in Y2H assays.	auxin biosynthesis, establishing the identity of the apical gynoecium.	
